# Anomalous quantization trajectory and parity anomaly in Co cluster decorated BiSbTeSe_2_ nanodevices

**DOI:** 10.1038/s41467-017-01065-7

**Published:** 2017-10-17

**Authors:** Shuai Zhang, Li Pi, Rui Wang, Geliang Yu, Xing-Chen Pan, Zhongxia Wei, Jinglei Zhang, Chuanying Xi, Zhanbin Bai, Fucong Fei, Mingyu Wang, Jian Liao, Yongqing Li, Xuefeng Wang, Fengqi Song, Yuheng Zhang, Baigeng Wang, Dingyu Xing, Guanghou Wang

**Affiliations:** 1 National Laboratory of Solid State Microstructures, College of Physics and Collaborative Innovation Center of Advanced Microstructures, Nanjing University, Nanjing, 210093 China; 2 Hefei National Laboratory for Physical Sciences at Microscale, University of Science and Technology of China, Hefei, 230026 China; 3 High Magnetic Field Laboratory, Chinese Academy of Sciences and Collaborative Innovation Center of Advanced Microstructures, Hefei, Anhui 230031 China; 4 Institute of Physics, Chinese Academy of Sciences, Beijing, 100190 China; 5 National Laboratory of Solid State Microstructures, Collaborative Innovation Center of Advanced Microstructures, and School of Electronic Science and Engineering, Nanjing University, Nanjing, 210093 China

## Abstract

Dirac Fermions with different helicities exist on the top and bottom surfaces of topological insulators, offering a rare opportunity to break the degeneracy protected by the no-go theorem. Through the application of Co clusters, quantum Hall plateaus were modulated for the topological insulator BiSbTeSe_2_, allowing an optimized surface transport. Here, using renormalization group flow diagrams, we show the extraction of two sets of converging points in the conductivity tensor space, revealing that the top surface exhibits an anomalous quantization trajectory, while the bottom surface retains the 1/2 quantization. Co clusters are believed to induce a sizeable Zeeman gap ( > 4.8 meV) through antiferromagnetic exchange coupling, which delays the Landau level hybridization on the top surface for a moderate magnetic field. A quasi-half-integer plateau also appears at −7.2 Tesla. This allows us to study the interesting physics of parity anomaly, and paves the way for further studies simulating exotic particles in condensed matter physics.

## Abstract (web summary)

The topological surface states usually appear in pairs in a topological insulator, with one on the top surface and the other on the bottom surface. Here, Zhang et al. utilize Co cluster to induce a Zeeman gap on one surface through antiferromagnetic exchange coupling, and observe a quasi-half-integer plateau, suggesting the parity anomaly of Dirac fermions.

## Introduction

In three-dimensional topological insulators (TIs), the nontrivial topology of their electronic bands causes a gapless state on the solid surfaces^1–12^, which is associated with forbidden backscattering, spin helicity, and its possible application in low-dissipation electronics. Dissipationless TI edge devices based on the quantum anomalous Hall^13–16^ (QAH) effect and the quantum Hall^17–20^ (QH) effect have been reported with clear quantization plateaus spaced apart by *e* ^2^ */h*, where *e* is the electron charge and *h* is the Planck constant. QH devices have been demonstrated to operate at more tolerable temperatures of almost 50 K, in which the surface electronics operate in a dimension among the (−3/2, −3/2) ~ (3/2, 5/2) spaces. Protected by time-reversal symmetry, practical TI nanodevices have a pair of parallel-transport carrier states on their two surfaces. However, due to the no-go theorem^21, 22^, the two topological surface states (TSSs) always appear as a pair and are expected to quantize synchronously.

Theoretically, breaking the time-reversal symmetry of a single Dirac fermion gives rise to a mass. The massive Dirac fermion has been predicted to exhibit a parity anomaly^2, 23, 24^ in high energy physics, although this phenomenon has yet to be observed experimentally despite being studied at some length in theoretical terms. The term “parity anomaly” implies that an infinitesimal mass will inevitably break the parity symmetry, resulting in a half-QH effect, which in turn means a half-QH conductance (1/2 *e* ^2^ */h*). The TSS whose low-energy effective spectrum mimics the Dirac fermion provides a perfect platform for achieving the parity anomaly. However, asynchrony quantization has still not been achieved to date, and the quantization trajectory of a single surface has not yet been studied.

Here we describe the deposition of Co clusters on bulk-insulating BiSbTeSe_2_ devices, which delays the Hall quantization of the top surface. An interesting quasi-half-integer plateau (−3/2 *e* ^2^ */h*) appears at −7.2 Tesla (T). Renormalization group flow diagrams^14, 25^ (RGFDs) allow extraction of two sets of converging points (CVPs) in the (*σ*_xy_, *σ*_xx_) space, where the top surface exhibits an anomalous quantization trajectory, while the bottom surface retains the 1/2 quantization. This effect can be interpreted using a delayed Landau level (LL) hybridization (DLLH) model, in which the Co clusters induce a sizeable Zeeman gap ( > 4.8 meV) through antiferromagnetic exchange coupling on the top surface.

## Results

### The QH effect of the surface-dominated TI

**Fig. 1:**
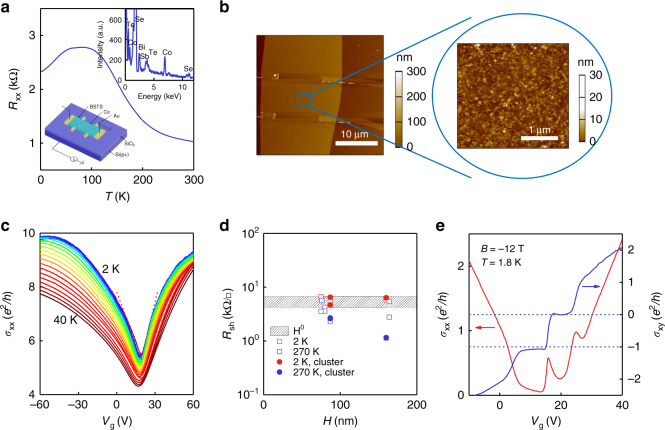
Surface dominant transport and quantum Hall effect in the BSTS device. **a** A typical curve of temperature-dependent resistance. The inset shows the backgate measurement configuration and the elemental composition of the sample. **b** Atomic force microscopic image of the device. The zoom-in of (**b**) shows the granular morphology of the sample surface. The sizes of the clusters are in the tens of nanometers and their heights are about 5 nm. The scale bar on the left is 10 μm (1 μm in the zoom-in). **c** Gate-dependent conductivity from 2 to 40 K with a step of 2 K, exhibiting the bipolar characteristic. The minimum conductivity appears at a gate voltage of around 16.6 V. **d** The sheet resistance of several samples (some of them measured before the Co cluster deposition). Red dots are the data measured at 2 K, all of which fall within a small range, with or without Co clusters. This indicates the dominance of the surface transport. The blue dots, measured at 270 K, fall outside the shadowed regime, however. *H* is the height of the samples, and *H* ^0^ denotes the zeroth power of *H*. **e** Quantum Hall effect observed at a temperature of 1.8 K and a field of −12 T

We fabricated field-effect-transistor BSTS^10^ devices using a standard lift-off procedure (see “Methods” section). The Co clusters were deposited on the bare devices using a cluster beam source as described elsewhere^26^. A schematic of our devices is shown in the inset of Fig. 1a. Elemental energy-dispersive X-ray spectroscopy was used to confirm the successful deposition of the clusters. We measured the sample both before and after Co cluster deposition; we refer to the sample after Co deposition as sample A, and the sample before Co deposition as sample A′. Figure 1a also indicates the typical temperature (*T*)-dependent resistance of sample A, showing insulating behavior at high temperatures (above 100 K), which is attributed to the transport of the bulk electronic state, followed by a metallic increase at low temperatures. This is similar to the case for pristine BSTS as reported by other groups^11, 17^. The pristine bulk maximum resistivity reached >10 Ω cm. An atomic force microscopic image (Fig. 1b) shows small islands with diameters of a few tens of nanometers and heights of around 5 nm. We measured several samples, and we list all the transport parameters of the devices in Supplementary Table 1.

In Fig. 1c, we show the backgate voltage (*V*_g_)-dependent longitudinal conductivity (*σ*_xx_) of sample A measured at a series of temperatures from 2 to 40 K at intervals of 2 K. All the transfer curves show bipolar transport behaviors, where the minimum conductivity corresponds to the Dirac point (DP) of the bottom surface. At *T* = 2 K, the DP is around *V*_g_ = 16.6 V and the minimum conductivity is about 5 *e* ^2^ */h*. Furthermore, *σ*_xx_ exhibits a linear dependence on gate voltage^18, 27^. We measured several samples, including some prior to cluster deposition. We show the sheet resistance (*R*_sh_) of all of these at 2 K (red dots) and 270 K (blue dots) in Fig. 1d, where we find that all the values of *R*_sh_ at 2 K fall into a small range independent of their thickness. Here, *H* is the height of the samples. This implies surface dominant transport in our devices, allowing successful observation of the QH effect.

High magnetic field drives BSTS to the QH state. In Fig. 1e, at a magnetic field (*B*) of 12 T, the Hall conductivity (*σ*_xy_) of sample A steadily reaches zero and −*e* ^2^ */h* while scanning the backgate voltage from 0 to 40 V. In the meantime, the longitudinal conductivity (*σ*_xx_) fluctuates and shows local minimums at both Hall plateaus. Similar QH effects^17–20^ have been interpreted based on the fact that the two TSSs (top and bottom) each contribute a half-integer plateau. The DP for the bottom surface is *V*_g_ = 16.6 V (Supplementary Table 1). Hence, we assign a half-integer index *ν* for each surface for both plateaus^17, 18, 20^. *ν*_b_ (bottom surface) is 1/2 and −1/2, respectively, and *ν*_t_ (top surface) is maintained at −1/2. The top surface is p-type transport.

### The anomalous quantization trajectory extracted by RGFD

**Fig. 2:**
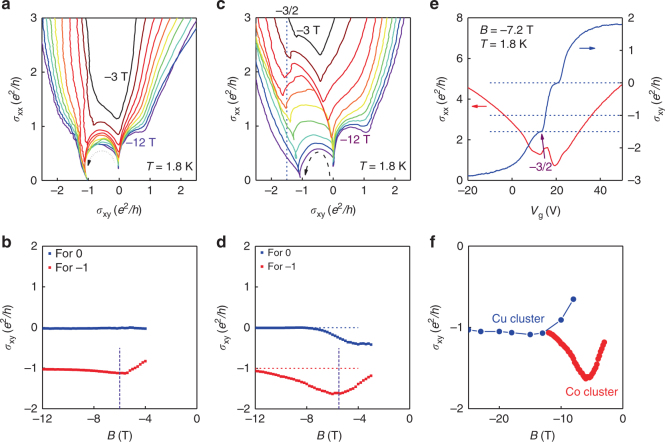
Renormalization group flow diagram analysis with the result of quantization trajectory of single Dirac channel. **a** Renormalization group flow diagram (RGFD) analysis in (*σ*_xy_, *σ*_xx_) space based on the data measured between −3 and −12 T with the step being −1 T for the sample before the Co deposition (sample A′) at 1.8 K. The converging points (CVPs), the local minimum with a vanishing *σ*_xx_ and plateau *σ*_xy_, indicate complete quantum Hall (QH) filling. **b** We note two sets of CVPs, pointing to *σ*_xy_ of 0 and −*e* ^2^ */h*. The dashed line marks the *σ*_xy_ minimum. **c** RGFD analysis in (*σ*_xy_, *σ*_xx_) space based on the data measured between −3 and −12 T with a step of −1 T for the Co-decorated sample (sample A). Note the anomalous RGFD trajectory is characteristic in this system. **d** The CVP trajectories for sample A. The converging trajectories are clearly different. The dashed line marks the *σ*_xy_ minimum. **e** The 3/2 QH plateau observed at −7 T and 1.8 K, while scanning the gate voltage. **f** The CVPs of the −1 plateau for the Cu clusters decorated BSTS (sample D) and the Co clusters decorated BSTS (sample A). Sample D does not show the anomalous trajectory of sample A

For graphene, integer QH plateaus of series (4*n* + 2) *e* ^2^ */h* have been observed^28, 29^, due to the spin and sublattice degeneracy of the material^30–32^. QH experiments on TI devices suffer from parallel transport of the two surface states and result in QH plateaus of (*n*_T_ + *n*_B_ + 1) *e* ^2^ */h*. However, in our cluster-decorated device it is interesting to note that the quasi-half-integer −3/2 Hall plateau appears when measuring at a medium field of −7 T, as marked by the purple arrow in Fig. 2e, in which a zero plateau is also seen. Both plateaus are supported by fixed *σ*_xy_ values and local minimums of *σ*_xx_. We prepared three different samples. Measurements made on two other devices also show QH plateaus near −1/2 and −3/2 *e* ^2^ */h* (Supplementary Fig. 3 and Supplementary Note 2). The reproducibility for different samples indicates that the quasi-half-integer plateau should be independent of both the parameter windows and the details of the samples. These observations lead to the question: Is this quasi-half-QH plateau an indication of the Landau quantization of a separated Dirac channel^2^? In the following, we devote our efforts to serious analysis of the nature of the half-integer plateau by extracting the RGFD of the corresponding state.

We note in Fig. 1e that when the magnetic field is increased to −12 T, the plateau of the sample becomes *n* = −1, corresponding to the quantization of both surfaces, *ν*_b_ = −1/2 and *ν*_t_ = −1/2. For the *n* = 0 state, *ν*_b_ = 1/2 and *ν*_t_ = −1/2 is well maintained^17, 20^. The context is better explained by sweeping the backgate voltage at a fixed magnetic field. RGFD analysis was carried out in the (*σ*_xx_, *σ*_xy_) space before (Fig. 2a, b) and after cluster deposition (Fig. 2c, d). The CVPs of the RGFD indicate complete Landau quantization of the bottom surface. Two sets of CVPs can be seen with *σ*_xy_ pointing to 0 and −*e* ^2^ */h*, respectively. Clearly, both sets of CVPs show continuous evolution and finally reach an integer with increasing field. Note that in all states of the CVPs in RGFD, the bottom surfaces have been quantized. In the whole parameter region, it is the deposited top surface that demonstrates a continuous evolution of Hall conductivity. Therefore, the −3/2 QH plateau should be the result of the sum of two contributions, with one being the −1/2 quantized bottom surface and the other being the −*e* ^2^ */h* Hall conductivity from the top surface.

In Fig. 2d, we plot the two sets of CVPs as curves, which are essentially the quantization trajectories of the top surfaces after subtracting a −1/2 (or 1/2) integer from the QH index of the bottom surfaces^17^. This way, we can extract the quantization trajectory of a separate Dirac channel. As shown in Fig. 2d, the quantization of the −1 plateau travels along an anomalous trajectory over a large range of nearly one *e* ^2^ */h*, which is not seen in the undecorated BSTS (Fig. 2b). We repeated these measurements in two other samples, where the quantization trajectories were anomalous (Supplementary Fig. 3 and Supplementary Note 2) and reproduced in both cases. Being sample-independent, we expect such an anomalous quantization trajectory to be a characteristic of our Co-cluster-decorated BSTS samples.

## Discussion

We propose a DLLH model to interpret the anomalous LL quantization trajectory in Co-cluster-decorated BSTS, in which two effects are taken into account. First, the Co clusters form some magnetic moments with finite magnetization strengths *M*_z_(*B*) under a field. The magnetic moments of the Co clusters further polarize the Dirac fermions on the top surface through antiferromagnetic coupling, which results in a sizeable Zeeman gap^19, 33^ *m* = −*JM*_Z_/2*μ*_B_, where *J* is the exchange interaction constant and *μ*_B_ is the Bohr magneton. Second, a medium field enlarges the Zeeman gap and forms the LL quantization. Solving the Dirac equation with a gauge field (Supplementary Note 3 and 4), we obtain an unexpected zeroth LL with energy *E*_0_ = −sgn(*B*)*M*_Z_/2*μ*_B_, which is positioned precisely at the top of the Zeeman gap. Such a magnetization condition is reasonable in our sample. Here the magnetization of the top surface increases with the field, therefore it is hard to detect directly and hard to see any loops. However, we may check its effect by fitting a weak antilocalization using the Hikami–Larkin–Nagaoka formula. We find that the channel parameter decreases when we increase the fitting range of the magnetic field from (0–0.2 T) to (0–1 T). Such a decrease can be related to the contribution of weak localization from a magnetization gap of several meV.

**Fig. 3:**
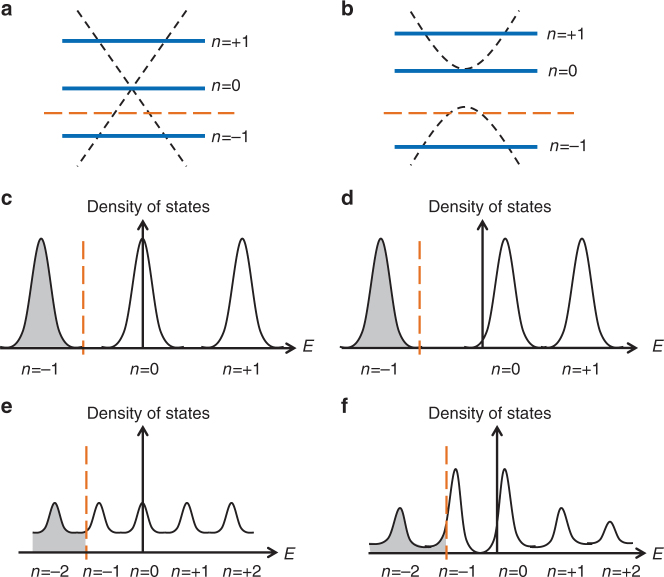
The delayed Landau level hybridization model. **a**, **c**, **e** and **b**, **d**, **f** schematically show the Landau Level (LL) diagram before/after Co cluster deposition, respectively, during Hall quantization. The orange dashed lines indicate the Fermi energy. Under a magnetic field, the deposited Co clusters induce a Zeeman-like gap in the Dirac cone of the top surface. The zeroth LL is shifted to the top of the Zeeman gap (**b**), while the bottom surface is unchanged, similar to the case for clean devices (**a**). This makes no significant difference at a sufficiently high field (**c**, **d**). At a medium field, it leads to the observed anomalous quantization trajectory (**e**, **f**). The anomalous RGFD trajectory (Fig. 2c) is the result of the delayed LL hybridization (DLLH) of the top surface (**f**)

We begin the interpretation by considering an undecorated sample in a sufficiently high field, where conventional Landau quantization is achieved in Dirac fermions (Fig. 3a, c). When the decreasing field reaches a threshold value, the LL spacings *d*_L_ gradually hybridize with each other (Fig. 3e), resulting in a smooth evolution of *σ*_xy_ from −*e* ^2^ */h* towards zero, as shown by the red curve in Fig. 2b. The threshold field can be interpreted from the position of the dashed line in Fig. 2d, with *B*_1_ = −6 T. The two effects discussed above take place after Co cluster deposition. The resulting shifted zeroth LL leads to an increase in the distance between −1 and 0 LL by *E*_0_. At a sufficiently high field, we can expect no significant difference between the pristine (Fig. 3c) and the deposited sample (Fig. 3d) because the LL spacing *d*_L_ is much larger than the Zeeman gap (Fig. 3b). This accounts for the perfect quantization at high field for both samples. However, at a lower field where the LL hybridization begins in the pristine case, the enlarged LL spacing *E*_0_ would be comparable to that in the LL spacing in the pristine sample. This results in the DLLH and leads to the following key difference between the two cases. In the pristine sample, Fig. 2b indicates that the −1 LL hybridizes before it crosses the Fermi energy. In the process of lowering the field, the electrons can still fill up all the extension states in the −1 LL, therefore no significant decrease in Hall conductivity is observed. However, in the decorated sample, hole states emerge in the −1 LL levels before hybridization, giving rise to the decrease of *σ*_xy_ before it evolves toward zero.

This is the experimental case, illustrating the physics of the anomalous quantization trajectory in RGFD (Fig. 2d). Around *B* = −7 T, the Hall conductivity from the bottom surface is still quantized at −1/2 *e* ^2^ */h* (Fig. 2b), while the top surface is non-quantized and exhibits Hall conductivity traveling along the anomalous trajectory due to the DLLH effect. Together with other effects such as the lateral cross section, the anomalous quantization trajectory leads to the intangible Hall plateaus observed in Fig. 2c, d. Finally, we note the results of some scanning tunneling microscopy experiments on undecorated TIs^34^, which show clear Zeeman shifts of the zeroth LL amounting to a few meV. However, this effect depends on the material. We find it appropriate to neglect the Zeeman effect for the undecorated case here (Supplementary Note 5).

On the basis of the above model, we estimate the width of the LLs as well as the Zeeman gap due to magnetic doping. It is known that the LLs begin to hybridize when their spacing becomes comparable to the width. Using this condition, we can estimate the LL width for the undecorated sample as *w* = 25.8 meV, by the critical field (−5.6 T) in the quantization trajectory (dashed line marked on the red curve in Fig. 2b). This broadening of the LL is related to the electronic inhomogeneity, which is most likely to originate from the random configuration of atoms in this material^35–37^. Figure 2d shows that the Hall conductivity begins to decrease from −1 when the field is reduced from 12 T, indicating that the electron gradually fills into the extension region in the *n* = −1 LL in this process. From *B* = −12 to −6 T, the Hall conductivity drops from −1 to −1.7 *e* ^2^ */h*, until which point the LL hybridization leads to an upturn in the Hall conductivity. We can use this observation to estimate the width of the extension region to be *d* ≈ 15.7 meV. Again using the condition for hybridization, we obtain *w*′ = *m* + 26.6 meV, where *w*′ is the LL width after deposition and *m* = *J*sgn(*B*)*M*_Z_/2*μ*_B_ is the Zeeman gap. Here the critical field moves to around −6 T (dashed line marked on the red curve in Fig. 2d) due to the delay effect and the broadened LLs. Usually, the extension region is much smaller than the width of the LL. As an estimate, we can safely assume that *d* < 0.5*w*′, and therefore *w*′ > 31.4 meV, such that *m* > 4.8 meV. This gives the lower bound for the estimated Zeeman gap.

To lend further weight to these results, we measured the sample with Cu cluster deposition. Different from the Co clusters studied here, the sample with a non-magnetic Cu cluster (sample D) does not exhibit an anomalous quantization trajectory (Fig. 2f); instead it shows a normal trajectory similar to the undecorated sample (Fig. 2a, b). For comparison, the CVPs for the Co-cluster-decorated sample (sample A) are plotted in Fig. 2f. The difference between Cu and Co cluster decoration can clearly be seen. We note that the non-magnetic clusters do not bring the effect to the QH state of the TI as the magnetic clusters do. The CVP trajectories of all the samples we measured are shown in Supplementary Table 1. The RGFD of sample D is shown in Supplementary Fig. 1 (Supplementary Note 1). The separate quantization of the top/bottom surfaces is thus further corroborated by the distinct LL widths. Nevertheless, the greatly disordered top channels still accommodate the QH transport, reflecting the priority of the spin-helical Dirac electrons.

## Methods

### Crystal growth and device fabrication

Well-refined BSTS crystals were grown by melting high-purity elements of Bi, Sb, Te, Se with a molar ratio of 1:1:1:2 at 850 °C for 24 h in evacuated quartz tubes, followed by cooling to room temperature over 1 week. The crystal has a low bulk carrier density, and can be cleaved easily. Following the work process of graphene, the BSTS microflakes were exfoliated and transferred on doped Si substrates coated with 300 nm SiO_2_. Au electrodes were applied by standard electron-beam lithography and electron beam evaporation. The Co clusters were deposited by a cluster beam system as described elsewhere^26^. The thickness of the samples and the clusters were measured using an atomic force microscope.

### Transport measurement and data analysis

Transport measurements were performed at low temperatures down to 1.8 K with a magnetic field up to 12 T. Standard lock-in amplifiers (Stanford Research System SR830) with a low-frequency (<20 Hz) excitation current of 200 nA (Keithley 6221) were used. The high magnetic field experiments were performed at the High Magnetic Field Laboratory, Chinese Academy of Sciences. The sheet resistance was *R*_sh* =*_ *R*_xx_ *(W/L) = ρ*_xx_, where *W* and *L* are respectively the channel width and length between the longitudinal magnetoresistance *R*_xx_ voltage probes. For the Hall effect, *R*_xy_ *= ρ*_xy_. The conductivities are *σ*_xx_ = *ρ*_xx_/(*ρ*_xx_^2^ *+ ρ*_xy_^2^) and *σ*_xy_ = *ρ*_xy_/(*ρ*_xx_^2^ *+ ρ*_xy_^2^). *σ*_xy_ and *σ*_xx_ are dependent on the parameters, such as temperature, magnetic field and gate voltage. The RGFDs are plotted with *σ*_xy_ on the abscissa and *σ*_xx_ on the ordinate in the (*σ*_xy_, *σ*_xx_) space. Here the temperature is unchanged, and the RGFDs are plotted with magnetic field for backgate voltage. Each curve in the RGFDs is in a fixed magnetic field, such as −12 T, so every point in the (*σ*_xy_, *σ*_xx_) space corresponds to a backgate voltage, which is actually the Fermi level.

### Data availability

The data in this work are available from the corresponding author on reasonable request.

## Electronic supplementary material

Supplementary Information
