# Supplementary material for: Anomalous quantization trajectory and parity anomaly in Co cluster decorated BiSbTeSe2 nanodevices
Source: Nat Commun. 2017 Oct 17;8:977. doi: 10.1038/s41467-017-01065-7 (PMC5645337; doi:10.1038/s41467-017-01065-7)
Supplement: Supplementary file 1 — Supplementary Information [file 41467_2017_1065_MOESM1_ESM.pdf]

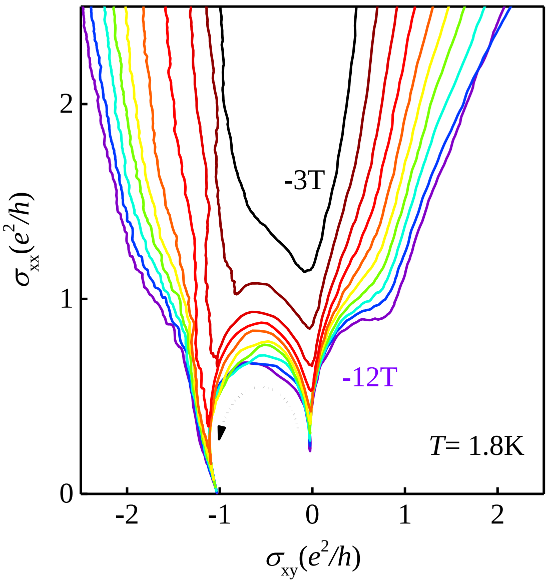

Supplementary Figure 1. The quantization trajectory of the Cu clusters decorated sample (sample D). The renormalization group flow diagram (RGFD) of the Cu clusters decorated sample. This RGFD analysis in (σ_{xy}, σ_{xx}) space is based on the data measured between at T = 1.8 K and high magnetic field. The converging points are plotted in Fig. 2f to compare with the Co cluster decorated sample (sample A). The sample parameters are listed in Supplementary Table 1.

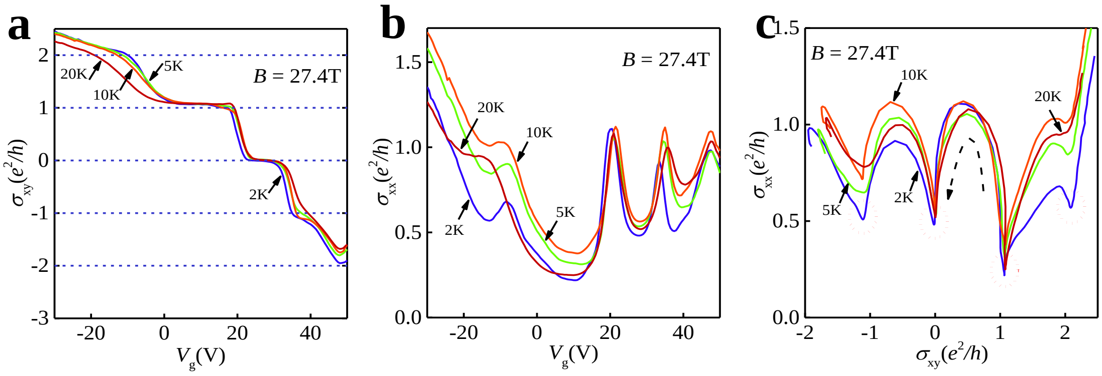

Supplementary Figure 2. Quantum Hall in Co cluster decorated Sample B and its RGFD analysis at magnetic field up to 27.4 Tesla. The backgate voltage dependence of the device conductance, where the Hall conductance (σ_{xy} (a)) goes quantized and the longitudinal conductance (σ_{xx} (b)) approaches zero with the increasing field strength. The quantum Hall (QH) states survive at the temperature of over 20 K. The σ_{xx} at the hall plateau is finite, the signature of the transport dissipation. (c) (σ_{xy}, σ_{xx}) are displayed with various gate voltages and temperatures, i.e., the RGFD. The dashed circles mark the successful Landau quantization.

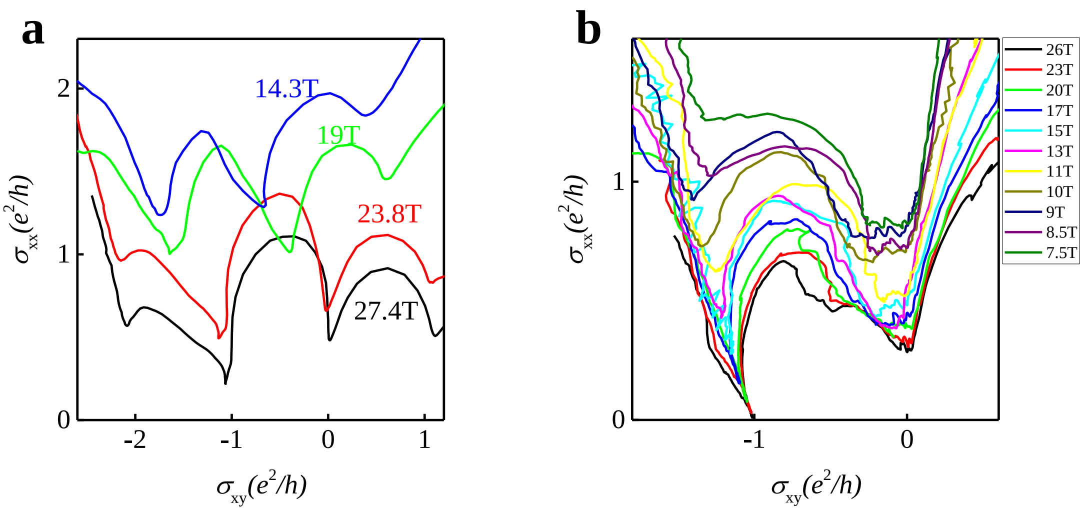

Supplementary Figure 3. Anomalous quantization trajectory for other samples and the RGFD. The RGFD of another Co decorated sample (sample C) (a). The trajectory of CVPs is plotted (b). Following the analysis in the main text, especially for Fig. 2c, we repeated the results which we discussed carefully for the anomalous quantization trajectory. Here, the anomalous RGFD trajectory is repeatable. These data are measured at T = 1.8 K.

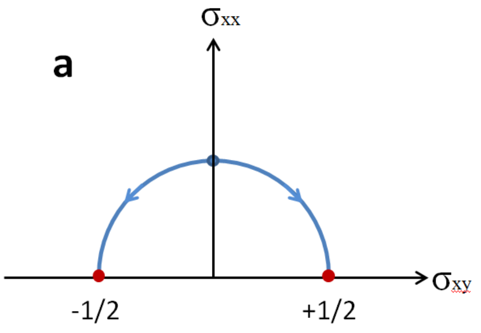

Supplementary Figure 4. The RG flow derived from the topological non-linear Sigma model.

Supplementary Table 1. Transport parameters of the devices.

Sample	Cluster	R_{sh} (2K)	σ_{min} (2K), V_g	Carrier type (V_g=0)	CVP Trajectory
A’	none	5550 Ω/□	4.0 e^2/h, 17 V	p	normal
A	Co	4668 Ω/□	5.1 e^2/h, 16.6 V	P	anomalous
B	Co	6369 Ω/□	4.0 e^2/h, 20 V	p	anomalous
C	Co	3907 Ω/□	4.0 e^2/h, 24 V	p	anomalous
D	Cu	4721 Ω/□	5.4 e^2/h, 15.5V	p	normal
E	none	5816 Ω/□	4.4 e^2/h, 20 V	p	normal

Note: Sample A’ is sample A before depositing Co cluster. A little difference in the V_g of charge neutral point between sample A and sample A’ is allowed in the experiment measurement. Three Co clusters decorated samples show anomalous quantization trajectory, while Cu clusters decorated and clean sample don’t have these phenomenon. The CVPs trajectories of these samples show clear dependence with magnetic clusters.

Supplementary Note 1. The Renormalization Group flow diagram of Cu cluster decorated sample.

Our conclusion can be more solid if similar experiments can be carried out on samples with different kind of clusters. Our cluster beam source is a Haberland-type source and can't generate magnetic clusters other than Co clusters. However, we can generate non-magnetic Cu clusters and carry out the experiments. This may figure out the real physics of the magnetic decoration.

Different from the Co decorated samples (Fig. 2d and Supplementary Fig. 3), here the RGFD of Cu clusters decorated sample (sample D) shows the normal trajectory, which is similar to the undecorated sample (Fig. 2a,b). The CVPs are plotted. As comparison, the CVPs for the Co clusters decorated sample (sample A) are plotted in the Fig. 2f. The difference between the Cu and Co clusters decoration can be clearly seen. It shows that the non-magnetic clusters don't bring the effect to the QH state of the topological insulator as the magnetic clusters do. We list the table to show the CVP trajectories of all the samples we measured in Supplementary Table 1. This is an evidence to confirm the crucial role of the magnetic clusters in our model.

Supplementary Note 2. Anomalous quantization trajectory for other samples and the RGFD.

In Supplementary Fig. 3, the CVPs clearly exhibit an anomalous trajectory like sample A in the main text. For the $-e^2/h$ plateau at -26 T of sample C, it travels over a lower plateau -3/2 e^2/h. And then its absolute value goes to a smaller value. The quasi-half-QH conductance can be seen from the RGFD in these samples. The trajectories of CVPs are plotted in Supplementary Fig. 3b. Note that the two samples need different magnetic field to achieve these results. Also the Dirac points are different from each other, although they are all in the p-type region without applying any back gate voltage. The magnetic field and carrier density windows are dependent on the details of each sample. However, the reproducibility means that the results in our experiments are not sample-dependent. The parameters of all the samples are listed in Supplementary Table 1.

Another problem is that at what extent the disorder will destroy the dissipationless transport. As we all known, the QH state has robustness against disorder, but too much disorder will destroy QH state. Here, we cannot find out the amount of disorder brought by the Co clusters. But considering that we have repeated and obtained the similar anomalous RGFD behavior in three independent samples, it is believed that the phenomenon observed here is not a coincidence but is indeed within an experimentally realistic parameter region. The main purpose of this work is focused on the separation of the quantization behavior of the top and bottom surface states. In order to do so, we have to introduce certain amount of disorder that can indeed

separate the two surface states, and on the other hand does not destroy the dissipationless transport. With regard to the question, i.e., under what parameter conditions will the QH behavior be preserved, is beyond the scope of the current work. This is an interesting topic that we wish to give quantitative answer in our further research.

Supplementary Note 3. Topological θ-term in the effective non-linear sigma model.

The robustness of quantized Hall conductance against disorder in the two dimension electron gas (2DEG) has been well studied by previous literature. The robustness and the renormalization group flow can be understood through an effective topological field theory around the mean-field saddle point, which is a non-linear sigma model with a topological θ-term. However, the effective field theory to explain the QH states in the TI devices is still in absence. To make our work as complete as possible, we now give a derivation of the topological field theory of the TI surface with disorder and under magnetic field, the result of which can explain the RGFD satisfactorily in our experiment.

In our experiment, we first investigated the undecorated sample under magnetic field. In this case, the Dirac fermion with a U(1) gauge field in the Landau gauge can be written as

$$H=v[\sigma_x k_x+\sigma_y(k_y-\frac{e}{c}By)], \qquad \text{(S.1)}$$

The corresponding Dirac equation can be solved which leads to the Landau levels (LLs) as

$$E_n=\pm v_F\sqrt{2\hbar eBn}/c \qquad \text{(S.2)}$$

where n is integer. There is a unique zero energy LL n = 0. It enjoys half of the degeneracy as the other LLs. Let us first only focus on the n = 0 LL by assuming that the Fermi energy lies in between n = 0 and n = 1. Then we consider a finite length in y direction with boundaries y = 0 and y = L, while setting the x direction to be infinite so

that k_x remains a good quantum number. Solving the Dirac equation in the finite size geometry, we formally obtain a chiral edge mode localized at the boundary, $y = 0$ and $y = L$. This edge mode is similar with but different from the chiral edge state in the QH state in the 2DEG as it only carries half of the quantized Hall conductance, i.e., $\sigma_{xy} = e^2/h$. Since we are only interested in the low energy infrared regime, the effective Hamiltonian of the chiral edge mode can be written as

$$H_{\mathrm{eff}}=\sum_{\mathrm{k_x,\alpha}} \alpha\, v k_{\mathrm{x}} c^{+}{}_{k_{\mathrm{x}},\alpha} c_{k_{\mathrm{x}},\alpha} \qquad \text{(S.3)}$$

where $\alpha = \pm$ denotes the $y = 0$ and $y = L$ boundaries respectively. Recalling the bulk-edge correspondence, the chiral edge mode here suggests a unique bulk topology characterized by the nontrivial Berry fiber bundle that leads to the Chern number[1] $C = 1/2$. On the other hand, it is known the quantum anomalous Hall (QAH) "Chern insulator" state of the gapped TI surface also enjoys the bulk topology with $C = 1/2$. Hence, we have arrived at the conclusion that both the $n = 0$ LL and the QAH state of the TI surface are associated with the bulk topology characterized by $C = 1/2$.

Now we are willing to consider the effect of disorder on the TI surface with magnetic field. It is known from the QH effect in the 2DEG that the fluctuation around the mean-field saddle point is usually described by a nonlinear sigma model in terms of the sigma field Q. Furthermore, the nontrivial bulk topology discussed above will give rise to a topological θ-term. For the 2DEG, the θ-term is found to be[2, 3]

$$S_{\theta}^{\mathrm{2DEG}}=-\frac{1}{8}\sigma_{\mathrm{xy}}\mathrm{Tr}Q[\partial_{\mathrm{x}}Q,\partial_{\mathrm{y}}Q]. \qquad \text{(S.4)}$$

From this topological θ-term, one can arrive at the RGFD in the low energy window. We now consider the corresponding topological θ-term correction due to the zeroth

LL in our undecorated sample. Since the zeroth LL shares the same bulk topology with the QAH state, we start with the QAH state in the TI surface. The gapped TI surface by breaking time-reversal symmetry can be understood as a Chern insulator with C = 1/2. As the topological θ-term in the non-linear sigma model of the Chern insulator can be derived naturally from dimension reduction[4], we first investigate the three dimensional (3D) Weyl cone,

$$H^{+}_{3D}=\sigma\cdot\mathbf{k} \tag{S.5}$$

where $\mathbf{k}=(k_x, k_y, k_z)$. The no-go theorem ensures that the Dirac cones come in pairs with opposite chirality, i.e., another Weyl fermion is implicit, with the Hamiltonian,

$$H^{-}_{3D}=-\sigma\cdot\mathbf{k} \tag{S.6}$$

Then we consider a white-noise random scalar potential induced by disorder $V(\mathbf{r})$, that satisfy $\langle V(\mathbf{r})\rangle=0$ and $\langle V(\mathbf{r})V(\mathbf{r}')\rangle=V^2(\mathbf{r}\text{-}\mathbf{r}')$. To deal with the disorder, we resort to the replica method and then integrating out the potential (average of the disorder), which leads to the Lagrangian density as

$$L=\phi_i^{+}(\omega\delta_{ij}+i\sigma\cdot\nabla\delta_{ij}+i0^{+}\tau_3^{r,a})\phi_j+\frac{V^2}{2}\phi_i^{+}\phi_i\phi_j^{+}\phi_j \tag{S.7}$$

where $\phi_i=[\phi_i^r,\phi_i^a]^T$ is the 2D Grassmann spinor with both the retarded and the advanced Grassmann component, and i, j denotes the i-th and j-th replica field. τ_3 acts in the retarded-advanced space and the repeated notations are summed. A similar Lagrangian associated with H^{-}_{3D} is implicit. The non-linear sigma model of the 3D chiral fermions has a Wess-Zumino-Witten (WZW) term[5] (corresponding to the nontrivial Chern number C which can be calculated by the Berry flux through a 2D surface that wraps the chiral fermion). The WZW term reads as

$$S^{\mathrm{WZW}}=\frac{\mathrm{i}\alpha}{128\pi}\int \mathrm{d}\tau \mathrm{d}^3x\epsilon^{\mu\nu\rho\sigma}\mathrm{tr}Q'\partial_\mu Q'\partial_\nu Q'\partial_\rho Q'\partial_\sigma Q' \quad \text{(S.8)}$$

where Q' is the extended sigma field, where $\alpha=\pm$ for $H^+_{3\mathrm{D}}$ and $H^-_{3\mathrm{D}}$ respectively. Then we couple a U(1) gauge field to $H_{3\mathrm{D}}$ with disorder. Taking into account the WZW term and requiring the gauge invariance of the action, one can find the following effective action around the mean-field saddle point.

$$S^{\mathrm{eff}+}_{3\mathrm{D}}=-\frac{\sigma_{xx}}{4}\int \mathrm{d}^3x\mathrm{tr}(\partial_i Q\partial_i Q)-\frac{1}{16\pi}\int \mathrm{d}^3x\,\epsilon^{jk}A_3\mathrm{tr}\big(Q\partial_j Q\partial_k Q\big)+\dots \quad \text{(S.9)}$$

without losing any generality, we have assumed the chiral gauge field is applied in the z direction with $\mathbf{A}=(0,0,A_3)$. The "..." denotes the WZW term which will be exactly cancelled by the action from $\mathrm{H}^-_{3\mathrm{D}}$ and therefore can be neglected. For the implicit Weyl cone $H^-_{3\mathrm{D}}$, the effective action $S^{\mathrm{eff}-}_{3\mathrm{D}}$ can be obtained similarly and it satisfies $S^{\mathrm{eff}-}_{3\mathrm{D}}=S^{\mathrm{eff}+}_{3\mathrm{D}}$. The chiral gauge field, which couples oppositely to the two Weyl cones, breaks the time-reversal symmetry, separating the Weyl nodes in momentum space along k_z. Moreover, the gapped region in between the two Weyl nodes can be viewed as a stack of 2-dimensional Chern insulator with C=1, whose topological θ-term can be obtained from $S^{\mathrm{eff}}_{3\mathrm{D}}=S^{\mathrm{eff}+}_{3\mathrm{D}}+S^{\mathrm{eff}-}_{3\mathrm{D}}=2S^{\mathrm{eff}+}_{3\mathrm{D}}$ by dimension reduction. Hence, the topological θ-term of the Chern insulator with C= 1/2 is a result of either $S^{\mathrm{eff}+}_{3\mathrm{D}}$ or $S^{\mathrm{eff}-}_{3\mathrm{D}}$, which describe the top and bottom surface in our model. Through dimension reduction, the topological θ-term from either the top and bottom surface reads as

$$S^{\mathrm{eff}}_{2\mathrm{D}}=-\frac{1}{16}\int \mathrm{d}^2x\,\epsilon^{jk}\mathrm{tr}\big(Q\partial_j Q\partial_k Q\big). \quad \text{(S.10)}$$

As has been discussed before, this topological θ-term also applies to the zeroth LL of the TI surface under magnetic field. It is a correction to the conventional QH states from higher LLs. Adding this correction to the conventional non-linear sigma model

of the QH states[2, 3], the total topological field theory with a θ-term reads,

$$\mathcal{L}_{\text{top/bot}} = -\frac{\sigma_{xx}}{4}\text{tr}(\partial_i Q \partial_i Q) - \frac{\sigma'_{xy}}{8}\epsilon^{jk}\text{tr}\big(Q\partial_j Q\partial_k Q\big). \tag{S.11}$$

where $\sigma'_{xy}=\sigma_{xy}-1/2$.

The Larangian density for the top and bottom surface $\mathcal{L}_{\text{top/bot}}$ enjoys the same mathematical form with that in the QH states of the 2DEG[2], despite the undressed σ_{xy} being modified to σ'_{xy} as a result of the unconventional zeroth LL. Following the same route as in the 2DEG case, by integrating out the fast mode step by step, we can obtain a series of the coarse-grained Larangian density with a gradually lower energy scale. The renormalization of (σ_{xx}, σ_{xy}) in this process gives us the RGFD with the energy scale being reduced. From the results in the 2DEG, two fixed points must occur in a complete period of σ_{xy}, i.e., $(\sigma'_{xx},\sigma'_{xy})$ = (0, 0) and (0, 1). The addition correction due to the zeroth LL in our experiment further generates the new fixed point, $(\sigma'_{xx},\sigma'_{xy})$ = (0, -1/2) and (0, 1/2), for both the top and bottom TI surface. The RG flow in the low energy window is obtained and plotted in Supplementary Fig. 5a. The two red solid points represent the obtained two stable fixed points. The arrow denotes the flow direction with the energy scale being lowered. There is another unstable fixed point lying at σ_{xy}=0 and finite σ_{xx}.

Supplementary Figure 4a is the RGFD predicted by our nonlinear sigma model investigation of the pristine sample for either the top or the bottom surface. We note that there are two differences between the theoretical RG flow and the experimental setup. First, the RGFD obtained here describes the flow with changing the energy scale (scaling parameter), whereas, in experiment, it is the gate voltage V_g that is

varying. Second, in experiment, the conductance from both the top and bottom surface cannot be separately measured, and we should consider the sum of the two transport channel. To make the theory in agreement with our experiment, we note that the gate voltage of the top surface is fixed (between the -1 and 0 LL) while that of the bottom surface is tuned (from above the 0 LL to below the 0 LL). Hence, in low temperature regime, the top surface always stay at the fixed point $\sigma_{xy} = -e^2/h$, whereas, the bottom surface is driven from the fixed point $\sigma_{xy} = e^2/h$ to $\sigma_{xy} = -e^2/h$. Then the V_g-driven flow of the sum of the two surfaces can be obtained, as is shown in Supplementary Fig. 4b. This accounts for the experimental data (Fig. 2c) satisfactorily.

Supplementary Note 4. The exchange coupling enhanced Zeeman effect and the LLs in the deposited sample.

In the deposited sample, the local magnetic moments of the Co clusters will be magnetized by the field to form a long-ranged order. The net average moment antiferromagentically couples to the electrons in the TI surface via the exchange term, $V_{ex}=Js_zM_z/\mu_B$, where $J>0$. Inserting this additional term into the Dirac Hamiltonian of the top surface, we have

$$H(k)=\hbar v\sigma\cdot\mathbf{k}-\mu+m\sigma_3 \qquad \text{(S.12)}$$

where $m=JM_z/2\mu_B$ is the mass gap opened due to the exchange coupling. For large magnetic field, we have to consider the Landau quantization. Coupling the U(1) gauge field to the gapped Dirac Hamiltonian through $\mathbf{k}\rightarrow\mathbf{k}'=\mathbf{k}-e\mathbf{A}$. We take the Landau gauge $\mathbf{A}=(-By,0,0)$ and further define the creation and quantization operators,

$$a=\frac{1}{\sqrt{2eB}}(k_x'+ik_y') \qquad \text{(S.13)}$$

$$a^+=\frac{1}{\sqrt{2eB}}(k_x'-ik_y') \qquad \text{(S.14)}$$

They satisfy $[a,a^+]=1$. Solving the corresponding eigenvalue equation, we can obtain the LLs for $|n|\geq1$,

$$E_n=-\mu\pm\sqrt{2ev^2B|n|+m^2}, \qquad \text{(S.15)}$$

together with the zeroth LL,

$$E_0=-\mu+J\mathrm{sgn}(B)M_z/2\mu_B. \qquad \text{(S.16)}$$

Since M_Z is the magnetization strength induced by the field B, it has the same direction with the field and $\mathrm{sgn}(B)M_Z>0$. For a antiferromagnetic coupling $J>0$. The energy of the zeroth LL is $E_0=-\mu+m$ with $m>0$. $-\mu$ is the position of the Dirac point,

therefore the zeroth LL lies exactly at the top of the Zeeman gap.

Supplementary Note 5. Reasons for ignoring the Zeeman effect in the absence of Co clusters.

In our explanation of the anomalous RGFD, the Zeeman effect in the absence of Co clusters is ignored. This is based on our experimental observation. In our experiment, we have measured and obtained the renormalization group flow diagram (RGFD) for both the decorated and undecorated sample, where the anomalous quantization trace is found to exist only in the decorated sample. According to our analysis, a significant Zeeman gap will lead to the suppression of hybridization of neighboring LLs, and will result in the anomalous quantization trace. However, no anomalous quantization trace occurs in the undecorated sample, which suggests that the Zeeman effect in the undecorated sample should be insignificant.

Although the Zeeman gap in the Bi_2Se_3 and Sb_2Te_2Se has been measured[6] to be around several meV. We still expect that the Zeeman gap in BSTS could be much smaller. This is due to the following reasons.

First, the Zeeman gap under magnetic field for the undecorated sample reads as

$$m = g_s \mu_B |B|/2, \qquad \text{(S.17)}$$

where g_s is the Lande g-factor. The g-factor of topological surface state is still a highly debated question. It is reported[6] that the estimated g-factor in Bi_2Se_3 and Sb_2Te_2Se is +18 and -6 respectively. However, it is worthwhile to note that the g-factor of the topological surface state is completely different from that in the bulk and is highly material dependent. The different elements will induce different orbital characters of the wave function of the surface state, leading to completely different

g-factors. Hence, even though Bi_2Se_3 and Sb_2Te_2Se have been investigated[6], the *g*-factor of the material BSTS studied in our work is still unknown. To be more comprehensive, we can resort to some relevant works. In the study of the shift of zeroth LL[7] in Sb_2Te_3, through the experimental scanning tunneling spectrum, it is found that, for the undecorated material, the zero-mode deviation at 7 Tesla is at most around 1 meV, which is relatively small and neglected compared to the exchange term due to magnetic decoration. On the other hand, while studying the quantum oscillation in topological surface state[8] of Be_2Te_2Se, it is found that the measured n-$1/B$ curve does not show any significant deviation from a straight line, justifying that the Lande *g*-factor should be no larger than 2. The small *g*-factor leads to about 1meV shift of the zeroth LL due to magnetic field. More importantly, in $BiSbTeSe_2$, it has shown that there is also no obvious shift of the zeroth LL[9]. Therefore, this is a strong suggestion that the *g*-factor in BSTS may be much smaller than that in Bi_2Se_3 and Sb_2Te_2Se.

Second, from the experimental data of the RGFD, we can estimate the magnitude of the Zeeman gap due to Co decoration. The estimated magnitude has a safe lower bound which is 4.8meV. The actual Zeeman gap due to decoration could be much larger than the lower bound. Therefore, the Zeeman effect after decoration should be much more significant than that in the bottom surface (without decoration), which is around 1meV (or 3meV at most).

Due to the above reasons, we do not take the Zeeman gap in the bottom surface into account, which is believed to be not important to account for our experiment.

Supplementary References

1. Thouless, D. J., Kohmoto, M., Nightingale, M. P. & den Nijs, M. Quantized Hall conductance in a two-dimensional periodic potential. *Phys. Rev. Lett.* **9**, 405 (1982).
2. Levine, H., Libby, S. B. & Pruisken, A. M. M. Electron delocalization by a magnetic field in two dimensions. *Phys. Rev. Lett.* **51**, 1915 (1983).
3. Khmel'nitskii, D. E. Quantization of Hall conductivity. *Pis'ma Zh. Eksp. Teor. Fiz.* **38**, 454 (1983) [*JETP Lett.* **38**, 552 (1983)].
4. Zhao, Y. X. & Wang, Z. D. Disordered Weyl semimetals and their topological family. *Phys. Rev. Lett.* **114**, 206602 (2015).
5. Pruisken, A. M. M. On localization in the theory of the quantized Hall effect: A two-dimensional realization of the θ-vacuum. *Nucl. Phys. B* **235**, 277 (1984).
6. Fu, Y. S. *et al.* Observation of Zeeman effect in topological surface state with distinct material dependence. *Nat. Commun.* **7**, 10829 (2016)
7. Jiang, Y. *et al.* Mass acquisition of Dirac fermions in magnetically doped topological insulator Sb_2Te_3 films. *Phys. Rev. B* **92**, 195418 (2015)
8. Xiong, J. *et al.* High-field Shubnikov–de Haas oscillations in the topological insulator Bi_2Te_2Se. *Phys. Rev. B* **86**, 045314 (2012)
9. Xu, Y. *et al.* Observation of topological surface state quantum Hall effect in an intrinsic three-dimensional topological insulator. *Nat. Phys.* **10**, 956 (2014)
